# Rheumatoid Arthritis

**DOI:** 10.1155/2013/958437

**Published:** 2013-11-28

**Authors:** Daniela Melchiorre, Anna Maria Iagnocco, Suchitra Acharya, Guglielmina Pepe, Marco Borderi

**Affiliations:** ^1^Department of Experimental and Clinical Medicine, Rheumatology, University of Florence, Florence, Italy; ^2^Department of Rheumatology, The Sapienza University of Rome, Rome, Italy; ^3^Bleeding Disorders and Thrombosis Program, Cohen Children's Medical Center of New York, NY, USA; ^4^Department of Experimental and Clinical Medicine, Critical Medical Care, University of Florence, Florence, Italy; ^5^Infectious Diseases Unit, University of Bologna, Bologna, Italy

Our team have worked together to ensure the best contribution and the greater impact of each accepted paper. Independent peer review remains a central part of the quality-control process applied to the publication of the scientific literature. All papers published are scrutinized by the editors, and those considered suitable are sent to two or three expert referees. In addition, each paper is allocated to a member of the editorial board specifically responsible for ensuring the adequacy of the peer-review process and providing an additional perspective regarding the quality of the paper. Papers are only accepted for publication after authors have satisfactorily addressed the issues raised by referees and the editors. Thus, in addition to peer review, key quality-control roles are exercised at multiple steps in the publication process by the editors to ensure the effectiveness, impartiality, and integrity of the peer-review and editorial processes. Furthermore, the six accepted papers, reported at the end, have tackled the topics of the special issue, for each of its arguments: (1) from molecular pathways to clinical trials; (2) predisease—what happens before diagnosis; (3) pain in rheumatic diseases and other arthropathies; (4) sonographic exam and clinical aspects in RA; (5) anti-TNF blockers and comorbidities in RA; (6) comorbidities in rheumatic diseases; rehabilitation and quality of life in RA; (7) muscle growth and atrophy in acute and chronic inflammation; (8) genetics and regulation of immune response; (9) bone and cartilage-regeneration—imaging. Some of them have opened up new areas for research in rheumatology. Others have developed a new point of view in regard to the etiopathogenesis, as the role of the hormones in the females or DNA damages, and the therapy in rheumatoid arthritis. Others have contributed to a better understanding of comorbidities that sometimes may be unsuccessful effective therapies if not properly considered. Everyone shall expand with their publication a more fruitful discussion on this rheumatic disease that is still disabling and seriously compromises the quality of life of patients who suffer. We have worked with great pleasure and being satisfied we conclude this special issue, hoping to repeat this challenging experience. Finally, we hope to have broadened, at least in part, together with the authors the knowledge and research in rheumatology.

The accepted papers are as follows.

1. “*Total knee arthroplasty considerations in rheumatoid arthritis,*” by J. R. Danoff et al.
2. “*Laryngeal manifestations of rheumatoid arthritis,*” by A. L. Hamdan and D. Sarieddine.
3. “*Restless legs syndrome as a comorbidity in rheumatoid arthritis,*” by J. A. Gjevre and R. M. Taylor Gjevre.
4. “*The role of simvastatin in the therapeutic approach of rheumatoid arthritis,*” by L. Cojocaru et al.
5. “*Lower serum androstenedione levels in pre-rheumatoid arthritis versus normal control women: correlations with lower serum cortisol levels,*” by A. T. Masi et al.
6. “*DNA damage in rheumatoid arthritis: an age-dependent increase in the lipid peroxidation-derived DNA adduct, heptanone-etheno-2*′*-deoxycytidine,*” by T. Hamasaki et al.

*Daniela Melchiorre* *Anna Maria Iagnocco* *Suchitra Acharya* *Guglielmina Pepe* *Marco Borderi*

